# Willingness-to-pay for sustainable beer

**DOI:** 10.1371/journal.pone.0204917

**Published:** 2018-10-05

**Authors:** Sanya Carley, Lilian Yahng

**Affiliations:** 1 School of Public and Environmental Affairs, Indiana University, Bloomington, IN, United States of America; 2 Indiana University Center for Survey Research, Bloomington, IN, United States of America; University of Vermont, UNITED STATES

## Abstract

Breweries across the country are investing in energy efficient and low-carbon brewing practices. Drawing insights from the sustainable consumption and ecological economics literature, this analysis evaluates whether consumers are willing to pay more for sustainable beer and what predicts the value of the premium. Based on a survey of beer consumers from across the U.S. that contained one of two willingness-to-pay exercises, we evaluate what respondent attributes are associated with a higher willingness-to-pay for sustainably brewed beer. We find that the majority of beer consumers are willing to pay more for sustainable beer. Consumers who are prepared to pay a premium tend to already pay more per unit of beer, are more aware of their purchasing behavior and the manner in which their consumption patterns may affect the environment, and pursue lifestyles based on professional advancement, helping the environment, and helping other causes.

## Introduction

The U.S. brewing industry is highly energy-intensive [1, 2, 3]. The industry is also expanding rapidly. Between 2005 and 2015, the U.S. expanded their craft breweries from about 1,300 to over 4,400, which is a growth of about 217 percent over a ten-year time span [4]. The average number of barrels produced per craft brewery has also increased over this time period at an average of over 12 percent per year [4]. Assuming that these remarkable growth rates will continue into the future, the industry will spend a great deal on energy and consume significant greenhouse gas (GHG) emissions.

There are many investments that breweries can make to enhance energy efficiency and reduce the carbon intensity of the brewing process, such as through the purchase of dynamic wort boiling systems, enhanced insulation, heat recovery, or alternative, distributed energy systems like solar or combined heat and power, to identify just a few [1, 2, 5, 6]. Several breweries have pursued these options, such as Allagash Brewing Company, which offsets all of its electricity consumption with the purchase of renewable energy credits; or New Belgium and Sierra Nevada, both of which have solar panels and an onsite wastewater treatment plant with methane capture; or Odell Brewing Company, which has insulated brewing vessels and steam recapture. The Brewers Association, the main association for craft breweries across the country, also regularly publishes sustainability manuals that outline how to implement sustainability practices and monitor and evaluate these efforts, such as the annual Sustainability Benchmarking Report. Also, an increasing number of breweries have sustainability webpages that outline all of the activities that distinguish the brewery in their environmental footprint (see, e.g., https://bearrepublic.com/sustainability/ or https://www.bellsbeer.com/sustainability).

But despite the apparent importance of sustainability in brewing, investment in energy efficient or low-carbon technologies will require breweries to spend significant amounts of money on upfront costs. These investments will also raise the price per unit of beer, at least in the short run, and potentially render the more sustainable, energy efficient breweries to be no longer cost-competitive with other companies; that is, unless beer consumers value beer as an eco-commodity and are willing to pay more for a beer made with sustainable practices. Evidence suggests that consumers may value sustainability in the making of a commodity such as beer, as the Brewers Association asserts, “Increasingly, environmental stewardship is a priority for beer drinkers, brewers and future generations. Maintaining a healthy balance between stewardship, social enrichment, and economic vitality is important to the future of craft brewing” ([7], p. 5). Studies have yet to confirm, however, that consumers would value such sustainability efforts in brewing and be willing to pay a premium for the resulting product.

This analysis studies whether consumers are willing to pay more for sustainable beer and what predicts consumers’ willingness-to-pay (WTP). It draws on a survey taken of beer consumers and purchasers from across the U.S. that contained one of two WTP exercises, administered randomly. In this analysis, we evaluate what respondent attributes are associated with a higher WTP for sustainably brewed beer, drawing on insights provided by the sustainable consumption and ecological economics literature.

This analysis makes three primary contributions to the literature. First, the extant literature on social sciences and beer, and more specifically beer consumption preferences and the factors that drive such preferences, is thin. We seek not only to inform this literature, but to help establish a foundation of knowledge in this nascent field. Second, although the literature on sustainable consumption is much more extensive, few analyses on the topic pull from such an extensive range of possible explanations for sustainable behavior; and no study does so in the context of sustainable beer. Third, this analysis imparts practical implications about individuals’ preferences for lower-carbon commodities that can be used to inform decision-making within breweries, as well as possibly extended to other industries and their sustainability decisions.

We begin with an overview of the relevant supporting literature and the beer industry. We then turn to the research design, present results, and discuss the implications of the results.

## Background

### Sustainable consumption and willingness-to-pay

This analysis is situated within the literature on sustainable consumption. This body of literature is characterized by studies that evaluate how and why individuals adopt more sustainable behaviors, what characteristics define the consumer of different green or eco-commodities, and what predicts whether a respondent values sustainable attributes and is therefore willing to pay a premium for those attributes. This literature generally operates on the premise that a transition toward more sustainable production will necessitate that consumers are willing to practice sustainable consumption and, as needed, be willing to pay for the added value of green commodities [8].

This literature has focused on high involvement environmental impacts such as household habits, food and perishable purchases, and transport purchase decisions and habits [9, 8]. Examples of related extant literature focus on technological eco-innovations [10] such as electric vehicles and solar panels [11], household commodities such as recycled paper or other green products [12, 9, 13, 14], green electricity [15], or perishable, sustainable products such as organic or fair-trade coffee [16, 17], organic food more generally [18, 19], and other types of groceries [9, 17]. If one also conceives of consumption as involving the full life cycle of a product, then this literature also extends to the topics of recycling [20] and transportation for food [21], among other domains. These articles consistently find that consumers are willing to pay a premium for a sustainable or eco-products; however, not all consumers are willing to pay more, nor are they all willing to pay the same amount. An important question then is what attributes define a sustainable consumer?

This literature tends to highlight in particular the importance of environmental attitudes and values in shaping sustainable consumption [22, 19, 23, 24, 25, 8, 9], or more generally serving as an antecedent to pro-environmental behavior. One of the more common set of measures employed in this literature to capture environmental attitudes and values is the New Environmental Paradigm [24, 25].

One’s views about the importance of protecting the environment or the fragility of our ecosystem, however, does not necessarily suggest that an individual places personal priority on such values. Yet, the literature underscores the importance of consumers’ personal sense of responsibility and accountability for environmental behavior as a distinct predictor of sustainable behavior [8]. When a consumer, for example, believes that his/her consumption behavior can make a difference—either for influencing producer decisions or for the environment more generally—this sense of consumer effectiveness has the potential to influence pro-environmental behavior [26]. Similarly, when a consumer believes that the onus of maintaining a clean environment rests with the government or private industry, or is the responsibility of others such as neighbors or peers, then he or she may be less likely to adopt sustainable consumption behavior, although on the other hand, an individual may be more likely to be a sustainable consumer when he/she observes that producers are making efforts, or when she/he observes peers’ sustainability behaviors.

An exclusive focus on environmental attitudes and norms assumes a high level of conscious thought and decision-making. The sustainable consumption literature points out, however, that many environmentally-related activities that consumers undertake are likely less deliberate and dictated by conscious thought but, rather, are more likely rooted in habit and part of a sequence of everyday activities [27, 28, 8]. Lifestyle theory posits that groupings of personal habits and practices in which one typically engages can reflect, as well as inform, an individual’s self-identity or self-concept [11]. Individuals with more environmentally-conscious lifestyles, and a self-identify rooted in sustainability, may be more likely to pay a premium for an eco-friendly product because consumption of that product is consistent with his/her self-identity.

Closely related to lifestyle theory is practice theory, or a recognition of the importance of specific and routinized behavior. Such practices of everyday life can demonstrate reflexivity, and may also lend insights about sustainable consumption behavior (see [29] for an overview of practice theory and related literature). Of course, practices or behavior can also serve as an expression of lifestyle and so these two concepts are closely interrelated. Those scholars that have considered sustainable consumption through the analysis of behavior tend to use measures of environmental and energy-specific behavior [22, 30].

Personal demographics are also important to sustainable consumption behavior, although some have found that they matter less so than aspects such as attitudes [9]. One example of a demographic attribute that is included in previous studies is the type of environment in which one lives. Whether one resides in a rural, urban, or suburban area may matter if, for no other reason, because location can hinder or facilitate access to other amenities. Type of surrounding environment can also affect one’s lifestyle, travel behavior, and personal compromises in life [8, 31].

Studies are inconclusive about gender. Analyses typically find that women are more environmentally sensitive then men [9, 13], but this sensitivity is not always evident in consumption behavior. Similarly, several studies find that income, education, and political orientation all predict environmental concerns (see [13] for a discussion of these studies) but there is not ample evidence that these demographic features are important determinants of WTP for a sustainable commodity.

While marital status is not generally a predictor of sustainable consumption, whether or not a household has children may be important. For example, Laroche and colleagues [9] find that those with children are more likely to pay more for a sustainable product (a finding reaffirmed in the case of potatoes by [32]). The authors speculate that this finding is likely due to enhanced sensitivity toward the environment out of concern that one’s offspring will suffer from future environmental damage. Another possible explanation for why parents might be willing to pay more for sustainable commodities is that consumption decisions are dictated by one’s life circumstances. In particular, those that work full-time and also raise children might experience more work-and-spend habits [33], and may also be more willing to pay for protecting the environment through commodity acquisition rather than through activist work or personal time devotion to an environmental cause.

### The beer industry and beer as a commodity

What makes beer an interesting commodity worthy of study? Sustainably brewed beer is distinctly different from other commodities such as organic food. Whereas organic food has the potential to protect human health directly, sustainably brewed beer is based on the production of the commodity and does not actually change the composition of the good nor affect human health positively or negatively. In this regard, sustainable beer is more akin to recycled paper or green cleaning products. Yet, there may be an additional distinction here as well. Whereas these products may be fundamentally different commodities than their non-sustainable counterparts (e.g., if recycled paper has a different texture than non-recycled paper or if green cleaning products are less tough on mildew than regular cleaners), presumably a sustainable beer and a normal beer should be identical. Thus, for those that are willing to pay a premium for sustainable beer, this premium is not a payment toward both health and the environment (e.g., organics), nor functional quality and the environment (e.g., green cleaning products), but is instead merely for the environmental aspects of the commodity. Of the topics covered in the extant literature, sustainable beer may be the closest to environmentally certified wood products (see, e.g., [34]) or eco-labeled electricity (see, e.g., [15]).

The market for beer is also a particularly interesting one, given a high degree of market segmentation among different types of production and consumer preferences. The domestic brewing industry is divided into four categories: brewpubs, microbreweries, regional craft breweries, and large domestic breweries. The first three types are typically considered “craft breweries” and are not only distinguished from their larger counterparts according to barrels produced but also according to their brewing techniques and social culture [35, 36, 37]. A brewpub is defined as that which sells at least 25 percent of its beer on-site. A microbrewery sells at least 75 percent of its beer off-site and produces at least 15,000 barrels of beer per year. A regional craft brewery sells between 15,000 and 6 million barrels of beer per year and is independently owned [7]. A large domestic brewery is one of mass-production, typically over 6 million barrels per year, and that makes predominantly traditional American lagers.

The beer industry in the U.S. is both highly consolidated and non-consolidated [38]. The industry is historically dominated by the largest breweries, such as MillerCoors and ABInBev, which have grown larger through the years due to consolidation [39]. These major industrial breweries still dominate the market, at over 70 percent of total production [40], but with a growing share of sales associated with craft breweries [35]. Over the last few decades, and particularly in the last decade, we have witnessed the “craft beer revolution” [36], in which demand for craft beer has soared and the market share of craft breweries has steadily risen.

The industry contains horizontally differentiated market segments among mass-produced domestic, imported, and craft beer products. Consumers within these markets are price inelastic, particularly those within the domestic market segment, and do not substitute commodities across the three market segments [36]; consumers, then, are highly segmented within market categories [35, 38]. Producers also advertise to these market segments differently and play strongly to brand personality [36]. Craft breweries tend to attract consumers through packaging and associated graphics and storylines, and work to cultivate a distinct social identity based on creativity, variety, quality, authenticity, and experimentation [35]. They also concentrate their advertising at breweries, festivals, social media, websites, and word of mouth [36, 37]. Many of these breweries use folklore, mythology, and the use of heroes in their branding [41]. Larger breweries, on the other hand, advertise through television, often during sports games, and grocery store displays such as beer towers [37].

Several analysts have observed that the craft beer revolution has occurred simultaneous to the public’s growing fascination with local consumption and more specialized food and drink products [36, 40]. Accompanying an interest in craft food is a willingness-to-pay more for creativity, variety, and quality. Analysts suggest that consumers of craft beer in particular eschew domestic mass-produced beer because it is perceived as too homogenous [41], and lacks variety or quality [36]. Craft beer is almost defiantly the opposite, with a range of over two dozen beer styles that one might find from any given brewery, and beer products offered from specific breweries as diverse—and perhaps bizarre—as pizza beer, lunar dust beer, avocado honey ale, coconut curry hefeweizen, or beard beer, to name just a few.

These insights from the beer industry literature suggest that consumers of mass-produced domestic beer may be fundamentally different than those of craft beer, and that one’s tastes and market segment allegiances should be accounted for when analyzing WTP for sustainable beer options.

## Research approach

### Survey design

This analysis employs an online survey design with embedded WTP questions. The survey was conducted through Amazon’s Mechanical Turk (MTurk), which is an online crowdsourcing workplace through which convenience samples may be acquired by recruiting workers to complete a survey. MTurk workers are registered with Amazon and opt into individual assignments. Given these terms, a common concern with MTurk surveys is the lack of representativeness of the population. Previous studies have analyzed the degree to which MTurk workers differ from population-based samples (see [42] for a review of this literature). These studies tend to find that MTurk respondents are, on average, younger, more highly educated, less affluent, less racially diverse, and more liberal than typical population-based samples. One study [42] compared an MTurk sample to the American National Election Studies 2012 Time Series Study and found, consistent with previous literature, that the samples differed in notable ways. Yet, these differences diminished after controlling for several easily-measurable demographic and political variables and, with such controls, MTurk can produce credible and generalizable results (see also [43]).

MTurk can also be used effectively to attract respondents of specific backgrounds or who exhibit specific characteristics of interest [44]. In our study, we sought to gather a sample of respondents who self-identify as beer consumers since it is the beer consumer population that is most likely to respond to brewing sustainability. Opt-in panels can also target specific populations by identifying possible respondents from panelist profiles and modeled indicators, but in the absence of a specific variable or reliable proxy for beer consumption with which to sample, screening for the characteristic of interest from a broad crowdsource like MTurk can be an efficient alternative. Thus, MTurk is an appropriate platform for our purposes.

**Fig 1:**
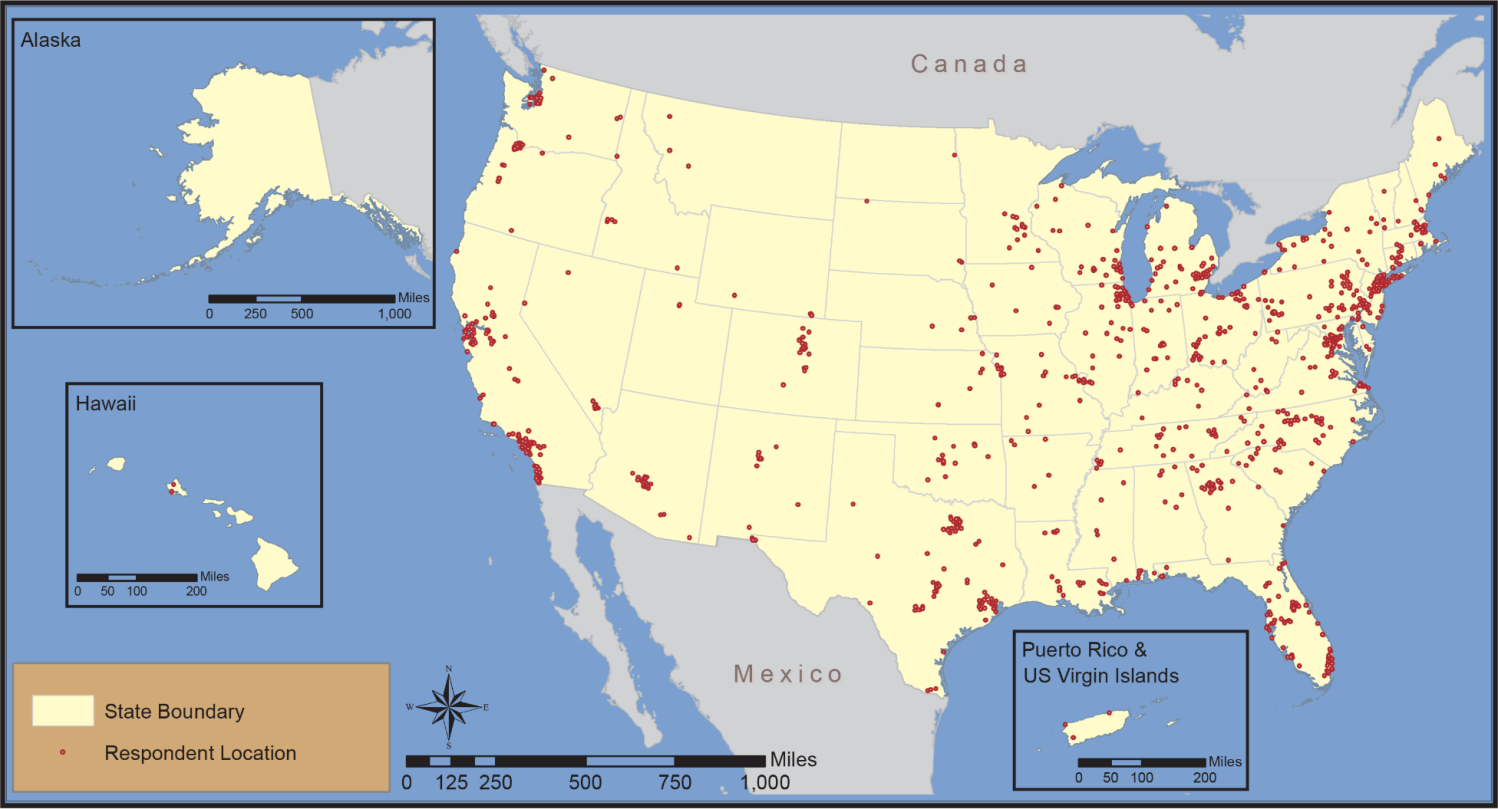
Study sample.

The survey sample was restricted to only those over 21 years of age who reside in the U.S., and that self-report as beer drinkers (consume at least one beer per month) and purchasers (sometimes, usually, or always is the one who purchases the beer that he/she consumes). The sample includes 1,095 respondents. Fig 1 maps where our respondents were located when they took the survey.

After two rounds of piloting the survey through a convenience and snowball sample, we administered the survey in October 2016. This time of year was carefully selected, since beer consumption has distinct season patterns [37] and it would not be ideal to sample during a period of time of low beer sales such as January or February, or particularly high beer sales such as mid-summer or holidays. October sales are fairly average relative to sales across all days of the year. Respondents were paid $1.20 per survey for an eight-minute survey, at a rate of $9 per hour.

The survey began with screening questions, then asked several questions about general beer preferences before proceeding to the WTP question. The next several sections of the survey asked about lifestyle activities and behavior, followed by questions about environmental perspectives and beliefs. The survey ended with questions about personal demographics. The survey instrument is presented in S1 File.

### Ethics statement

This research involved human subjects. It was approved by the Office of Research Compliance at Indiana University, under protocol number 1603199017. In accordance with this protocol, informed consent was provided by all study participants.

### Data

The dependent variable is the amount per ounce that a respondent would be willing to pay above the typical price of his/her favorite beer. We convert all responses to a per-ounce figure because different consumers purchase beer in different sized packages, from single bottles to 24-packs, mini kegs to quarter barrels, to all things in between: bombers, growlers, tallboys, crowlers, stovepipes, and more. We constructed two framing tracks that led to the WTP measure and randomly assigned respondents to one of the two treatments. One framed WTP within the context of an actual beer the respondent currently enjoys (“WTP1”) and the other within the context of a hypothetical beer designed by the respondent as part of a thought-exercise (“WTP2”).

In the WTP1 track, respondents were first prompted with: “Please think about a beer that you really like to drink. In the next few questions, we will ask you to describe a few things about this particular beer.” We then asked for the brand and name of the beer in an open-ended question, so as to ensure that the respondent had in mind a specific beer to use as reference, as well as the size package in which the beer is typically sold. For the latter, the response options ranged from a single beer to a 24-pack, with an “other” option in which respondents could enter the relevant answer. We next asked “Approximately how much is a [UNIT] of this beer? Please enter the price in dollars and cents,” where “UNIT” is filled in with the respondent’s reported size. This question gave us a baseline value for the typical amount that each respondent spends on his/her favorite beer.

To set up the WTP2 track, we first prompted the respondent with: “Please imagine a beer you would most enjoy drinking—think of it as your ‘ideal’ beer. In the next few questions, we will ask you to describe a few things about this beer.” We then asked the respondent to define his/her ideal beer by color, hop profile, and malt profile. For example, for the color question we asked, “For your ideal beer, rank the color from 1 to 10, where 1 is very light or pale and 10 is very dark. Please answer to the best of your ability, even if you think you don't know much about beer in general.” Response options included entering a number from 1 to 10 or checking a box for “Don’t care about the color.” We then asked the respondent to identify what size package his/her ideal beer would be sold in at the store, with the same response options as WTP1, and for any other details that s/he would like to tell us about his/her ideal beer. Then, to acquire a baseline value for the beer, just as we did with WTP1, we asked “If you found this exact beer on the shelf at a store, what is the most you would pay for a [UNIT] of it? Please enter this price in dollars and cents.”

Proceeding this set-up for both tracks, we provided the following information:

Many breweries across the U.S. are investing in equipment that helps them conserve energy or water, or use an electricity source that produces limited greenhouse gas emissions, such as solar panels. For consumers, these practices could make these beers more expensive, but with the benefit of saving energy and reducing greenhouse gas emissions.

Immediately following this information, we asked, “If [BEER] were brewed using such practices, would you be willing to pay more than [$VALUE] for a [UNIT] at a store?,” where “BEER” refers to either the respondent’s favorite beer that he/she entered in WTP1 or “your ideal beer” for those in WTP2; and “$VALUE” refers to the amount that each respondent reported that he/she would pay. For those that indicated that they would be willing to pay more, we followed up with, “How much more would you be willing to pay for a [UNIT] of this beer in addition to [$VALUE]? Please enter the maximum additional amount in dollars and cents.”

We used two tracks to detect difference in premiums that could potentially indicate the presence of hypothetical bias in at least one of the treatments. Hypothetical bias is the difference between the price a respondent claims s/he would pay on a survey question and how much s/he would actually pay. For instance, in estimations of WTP for remote public goods like nature conservation, there tends to be an overstatement of WTP, sometimes with a median overstatement of up to three times [45]. Although WTP measures of private goods may be less susceptible to overstatement than public ones since respondents may be motivated to *under*report to keep prices low, given that the premium in our study concerns an environmental good, we embedded the two tracks into the study design to detect differences. We hypothesized WTP1 would be equal or lower to WTP2, since the latter may contain more uncertainty than referencing an actual preferred beer for respondents. We found, however, no statistically significant difference in reported WTP between the two tracks.

We include several categories of independent variables, as drawn from the sustainable consumption literature reviewed above. First, the associated literature underscores the importance of environmental beliefs in shaping sustainable consumption behavior. To capture such beliefs, we construct several different measures. First, following the design in Barr and Gilg [22], we use a combination of New Environmental Paradigm (NEP) factors [24], revised NEP factors [25], and factors inspired by O’Riordan [46]. For each variable, we asked the respondent to rank the degree to which s/he agreed/disagreed with the statement, based on a 7-point scale. We combine these variables using principal component factor analysis with the rotational strategy varimax. We use the Kaiser-Meyer-Olkin to test the adequacy of using the factor loadings. Relevant details are outlined in Table 1. The environmental paradigm factors all load onto two factors. Second, we include a measure of the degree to which the respondent believes that his/her consumption behavior will impact the environment. The third variable measures the degree to which respondents believe that companies have the responsibility to make all of their products more environmentally-friendly.

**Table 1:** Principal component factor analysis results for environmental paradigm survey questions.

	Factor 1	Factor 2
**Factor 1: Delicate Environment and Resource Constraints (Cronbach's Alpha = 0.816; Eigenvalue = 3.14)**		
The balance of nature is delicate and easily upset.	0.7865	-0.1648
The Earth is like a space ship, with limited room and resources.	0.7323	-0.1749
Plants and animals do not exist primarily for human use.	0.6999	-0.2242
One of the most important reasons for conservation is to preserve wild areas.	0.7566	-0.0227
Exploitation of resources should be stopped.	0.7372	-0.1223
**Factor 2: Growth Limits and Human Science (Cronbach's Alpha = 0.709; Eigenvalue = 2.27)**		
There are no limits to growth for nations like the United States.	0.0128	0.6690
Modifying the environment seldom causes serious problems.	-0.3813	0.6165
Science will help us to live without conservation.	-0.2159	0.7566
Humans were created to rule over nature.	-0.4350	0.5840
Technology will solve many environmental problems.	0.0300	0.6385
Kaiser-Meyer-Olkin overall measure of sampling adequacy	0.855	

The second category of independent variables contains measures of lifestyle and behavior. The lifestyle practices are based on questions designed by Axsen and his colleagues [11] in their study on pro-environmental technologies. For each individual lifestyle practice, the respondent was asked to identify the frequency by which s/he engages in each activity, based on a 5-point scale. The behavior questions are specific to environmental practices, and are influenced by survey questions used in [30] and [22]. To extract this information, we asked respondents to mark how frequently they engage in each type of behavior, based on a 5-point scale. For both lifestyle and behavior variables, we employ principal component factor analysis. The lifestyle variables load onto four factors, as shown in Table 2: social and leisure, lifestyle practices aimed at the greater good, advancement, and sports and nature. The behavior questions all load onto two factors—green consumption and conservation/recycling—as displayed in Table 3.

**Table 2:** Principal component factor analysis results for lifestyle survey questions.

	Factor 1	Factor 2	Factor 3	Factor 4
**Factor 1: Social and leisure** (Cronbach's Alpha = 0.679; Eigenvalue = 2.17)				
Shopping	0.5167	0.4522	0.0866	-0.1307
Socializing with others	0.5287	0.2128	0.1151	0.337
Taking care of/spending time with family	0.49	0.4063	-0.1148	0.2504
Using the Internet for fun/leisure	0.7883	-0.196	0.0906	0.0637
Watching TV or movies	0.7964	-0.0181	0.0371	-0.0063
**Factor 2: Greater good** (Cronbach's Alpha = 0.575; Eigenvalue = 1.84)				
Helping the environment	-0.0307	0.5128	0.1974	0.4128
Religious or spiritual practices	-0.1506	0.7196	0.0342	0.0731
Volunteering or donating to charity	0.0212	0.6986	0.2399	0.1703
**Factor 3: Advancement** (Cronbach's Alpha = 0.585; Eigenvalue = 1.68)				
Developing career	0.1188	0.0399	0.6565	0.305
Researching or trying new technology	0.2333	-0.0098	0.7189	0.1324
School, lectures, other education	-0.104	0.2443	0.7571	-0.0593
**Factor 4: Sports and Nature** (Cronbach's Alpha = 0.510; Eigenvalue = 1.64)				
Playing sports, exercise, recreation	-0.0194	0.0033	0.1412	0.7784
Enjoying nature and the outdoors	0.1515	0.2455	0.0314	0.7213
Kaiser-Meyer-Olkin overall measure of sampling adequacy	0.738			

**Table 3:** Principal component factor analysis results for behavior survey questions.

	Factor 1	Factor 2
**Factor 1: Green consumption** (Cronbach's Alpha = 0.714; Eigenvalue = 2.38)		
Compost waste	0.6541	-0.0537
Use own bag at grocery store	0.6415	0.2617
Purchase organically grown food	0.7346	0.1486
Purchase recycled paper products	0.7342	0.2494
Buy from local stores	0.5518	0.251
**Factor 2: Conservation and recycling** (Cronbach's Alpha = 0.650; Eigenvalue = 1.86)		
Turn off the water faucet while brushing teeth	0.071	0.8361
Keep heating low to save energy	0.1705	0.808
Recycle	0.3548	0.5342
Kaiser-Meyer-Olkin overall measure of sampling adequacy	0.826	

We include a number of variables that characterize respondents’ beer preferences. The first variable also served as one of our screening questions—how frequently the respondent purchases beer, based on a Likert scale. The second question captures how often the respondent consumes beer with a continuous measure of the number of beers that the respondent consumed in the last week. The third variable is a dichotomous variable that reflects whether the respondent primarily purchases and consumes beer in his/her household versus in other places such as restaurants, breweries, or at social gatherings. A fourth variable helps us distinguish between consumers of craft beer versus mass-produced American lagers, a distinction that may be important given the significant evidence of market segmentation in the industry. To construct this variable, we asked respondents to identify which styles of beer they buy most often and allowed them to select up to three styles. For the variable of interest, we coded to equal one all of those respondents that *only* selected “Light-pale American lager,” and zero otherwise. In the regression analysis, therefore, the omitted categories include those that drink both domestic lagers and other varieties of beer, as well as those that exclusively drink non-domestic lager options. The final variable in this category accounts for how much the respondent already pays for her/his favorite beer, as converted to a dollars per ounce (oz.) figure, before considering whether to pay a premium for a sustainable version of the same commodity.

Finally, we include a set of personal demographics such as age, gender, education, political leaning, marriage status, household residency, and whether the respondent lives in a rural, urban, or suburban setting.

### Statistical approach

To determine which factors are related to greater WTP for sustainable beer, we estimate an ordinary least squared regression with robust standard errors. We confirm that the model does not suffer from multicollinearity by visually inspecting the pair-wise correlations among all variables as well as the variance inflation factor.

## Results

All variables are defined and presented with their descriptive statistics in Table 4. Out of the 1,094 respondents that answered the WTP for sustainable beer question, 642 (59%) said that they would pay more. The average response to the question was 1.8 cents/oz. This amounts to about 22 cents per 12-oz bottle of beer, or $1.30 per 6-pack of beer. The maximum amount that a respondent is willing to pay is $7.25 per 12-ounce bottle. These reported values are on top of the amount that the respondent already pays for his/her favorite beer, which is on average 14 cents/oz ($1.69/12-oz bottle).

**Table 4:** Variable definitions and descriptive statistics.

Variable	Description	Average	Std. Dev.	Minimum	Maximum
WTP for sustainable beer	Reported WTP for sustainable beer, in $/oz above the regular price	0.018	0.032	0	0.604
WTP1	Respondent was randomly sorted into the first WTP track	0.5	0.5	0	1
**Environmental Beliefs**					
Delicate environment and resource constraints	Factor 1 in Table 1	-7.52E-10	0.985	-4.63	2.13
Growth limits and human science	Factor 2 in Table 1	2.29E-09	1	-2.53	3.76
Consumption affects the environment	Degree to which respondent believes that his/her choices as a consumer will have a direct effect on the environment, based on 7-point Likert scale from 1 = strongly disagree to 7 = strongly agree	5.28	1.32	1	7
Companies are responsible	Degree to which respondent believes that companies have the responsibility to make all of their products more environmentally-friendly, based on 7-point Likert scale from 1 = strongly disagree to 7 = strongly agree	5.65	1.16	1	7
**Lifestyle and Behavior**					
Social and leisure	Factor 1 in Table 2	-6.04E-10	1	-4.57	2.2
Greater good	Factor 2 in Table 2	1.17E-10	1	-2.63	3.24
Advancement	Factor 3 in Table 2	5.44E-10	1	-2.85	2.86
Sports and nature	Factor 4 in Table 2	-1.35E-09	1	-3.8	3.1
Green consumption	Factor 1 in Table 3	5.72E-10	1	-3.02	2.74
Conservation and recycling	Factor 2 in Table 3	8.37E-10	1	-3.72	1.61
**Beer Preferences**					
Purchase frequency	Out of the beer that the respondent typically drinks, he/she purchases it: 1 = sometimes, 2 = usually, or 3 = always	2.3	0.668	1	3
Number of beers	Number of beers respondent reports consuming in the last week	7.95	8.75	0	100
House	Respondent most often drinks beer at his/her house	0.704	0.457	0	1
American lager consumer	Respondent reports liking American lagers and no other variety of beer	0.202	0.402	0	1
Price per ounce	The price per ounce that the respondent pays for his/her favorite beer	0.141	0.11	0	1.67
**Demographics**					
Age	Respondent’s age	34.56	9.94	21	74
Male	Respondent self-identifies as a male	0.565	0.496	0	1
Married or equivalent	Respondent is either married or living as married	0.491	0.5	0	1
Number in household	Number of individuals that live in the respondent's household	2.65	1.53	1	25
Political leaning	Respondent's political leaning on 7-point scale, where 1 = extremely liberal and 7 = extremely conservative	3.47	1.63	1	7
Education	Highest level of education completed, ordinal variable based on a 8-point scale where 1 = less than high school and 8 = doctoral degree	4.27	1.32	1	8
Income	Respondent's reported household income, ordinal variable based on a 10-point scale where 1 = under $15,000 and 10 = above $250,000	4.41	1.86	1	10
Rural	Respondent lives in a rural area (omitted category: suburban)	0.178	0.383	0	1
Urban	Respondent lives in an urban area (omitted category: suburban)	0.28	0.449	0	1

Table 5 compares the final sample with both a typical MTurk sample, as studied and documented by [47], and national averages. These estimates suggest that our sample of frequent beer consumers is more heavily male and younger on average than typical MTurk respondents and the national average, with more respondents in their 20s and fewer in their 30s and 40s. Education levels across the samples align fairly well, with slightly higher percentages of bachelor degree respondents in our beer consumer sample. The respondents match income status of typical MTurk respondents, are more likely to be single, and are more liberal than national samples but more conservative than a typical MTurk sample. Although our focus in the present paper is specifically on the beer consumer population, we also present results in Table A in S2 File in which we apply survey weights that more closely align our MTurk sample with a nationally representative sample.

**Table 5:** Respondent information relative to MTurk and national samples.

Demographic	Study MTurk Sample	Other MTurk Sample (Sheehan and Pittman, 2016)	National Probability Sample
Gender			US Census 2015
Male	56%	50%	49%
Age			US Census 2015
20–29	37%	20%	21%
30–39	38%	50%	19%
40–49	15%	20%	19%
50–75	10%	10%	41%
Education			US Census 2015
Less than high school	0.5%		11%
High school	9%	10%	29%
Some college	25%	25%	17%
2-year degree	11%	10%	10%
4-year degree	42%	35%	21%
Master's Degree	10%	15%	9%
Professional Degree	1%		1%
Doctorate	1%	5%	2%
Income			US Census 2015
Under $15,000	7%	<20%	12%
$15,000-$25,000	11%	>10%	11%
$25,000-$40,000		20%	15%
$25,000-$34,999	14%		10%
$35,000-$49,999	18%		13%
$50,000-$74,999	23%		17%
$40,500-$60,000		20%	15%
$60,000-$75,000		>10%	9%
$75,000-$99,999	14%	>10%	12%
$100,000-$149,999	9%	<10%	14%
$150,000-$199,999	2%	<5%	6%
$200,000-$249,999	1%	<5%	
$200,000+			6%
$250,000+	0.5%		
$300,000+		<5%	
Marital Status			US Census 2014
Never married, no children	43%	35%	
Never married			32%
Married, no children		12%	
Married with children		31%	
Married, includes separated	39%		52%
Living as married	11%	6%	
Divorced	7%	6%	10%
Widowed			6%
Other		10%	
Political Leaning			Election Results 2012 (Roper)
Voted for Obama in 2012		73%	51%
Voted for Romney in 2012		15%	47%
Voted for another candidate in 2012		12%	2%
			American National Election Studies 2012
Extremely liberal	10%		3%
Liberal	25%		10%
Slightly liberal	16%		11%
Moderate or middle of the road	23%		32%
Slightly conservative	12%		14%
Conservative	11%		18%
Extremely conservative	3%		4%
Don't Know/Haven't Thought			8%

Regression results are presented in Table 6. Models 1–5 isolate specific categories of independent variables and Model 6 combines all independent variables. For each category of independent variables, we report the percentage of total variation of the dependent variable explained by each category, as obtained through an R-squared decomposition from Model 6.

**Table 6:** Regression results with dependent variable WTP for sustainable beer (in $/oz).

	Model 1	Model 2	Model 3	Model 4	Model 5	Model 6	R2-decomposition
WTP Track							0.0817
WTP1	-0.000717					0.000931	
	(0.00196)					(0.00205)	
**Environmental Beliefs**							17.106
Delicate environment		-0.000824				-0.000872	
and resource constraints		(0.00132)				(0.00132)	
Growth limits and human		-0.000866				-0.00122	
science		(0.000905)				(0.000806)	
Consumption affects the		0.00364***				0.00187**	
environment		(0.000816)				(0.000760)	
Companies are		0.00287***				0.00150*	
responsible		(0.000839)				(0.000827)	
**Lifestyle and Behavior**							28.203
Social and leisure			0.000789			0.000715	
			(0.00130)			(0.00107)	
Greater good			0.00165			0.00211**	
			(0.00112)			(0.00104)	
Advancement			0.00148*			0.00146*	
			(0.000771)			(0.000831)	
Sports and nature			-0.000501			-0.000502	
			(0.000805)			(0.000854)	
Green consumption			0.00673***			0.00498***	
			(0.000981)			(0.00103)	
Conservation and			0.00239***			0.00119	
recycling			(0.000709)			(0.000802)	
**Beer Preferences**							45.796
Purchase frequency				0.000723		0.000984	
				(0.00137)		(0.00159)	
Number of beers				-0.0000627		-0.000136	
				(0.0000873)		(0.0000857)	
House				-0.00392**		-0.00322	
				(0.00194)		(0.00203)	
American lager				-0.00306*		-0.00153	
consumer				(0.00181)		(0.00209)	
Price per ounce				0.0776***		0.0739***	
				(0.0265)		(0.0270)	
**Personal Demographics**							8.813
Age					-0.000228***	-0.000185**	
					(0.0000786)	(0.0000785)	
Male					-0.00429**	-0.0000159	
					(0.00194)	(0.00212)	
Married or equivalent					-0.000464	0.000905	
					(0.00215)	(0.00206)	
Number in household					0.000729	0.000451	
					(0.000640)	(0.000630)	
Political leaning					-0.00204***	-0.000186	
					(0.000516)	(0.000639)	
Education					-0.000582	-0.00195*	
					(0.00105)	(0.00104)	
Income					0.000435	-0.000296	
					(0.000786)	(0.000807)	
Rural					-0.000556	0.000657	
					(0.00292)	(0.00255)	
Urban					0.000455	0.000442	
					(0.00209)	(0.00197)	
Constant	0.0182***	-0.0176***	0.0176***	0.00911*	0.0341***	0.00432	
	(0.00115)	(0.00563)	(0.000974)	(0.00482)	(0.00760)	-0.00971	
	1094	1061	1056	1094	1066	1000	

Standard errors in parentheses

* p<0.10.

* p<0.05.

*** p<0.01.

While the two environmental paradigm factors are not important predictors of WTP, we can observe the significance of degree to which respondents believe that they can have an impact on the environment through their consumption behavior and whether companies are responsible for offering sustainable products. These results suggest that it is less important that an individual holds generally environmentally-conscious beliefs than it is that he/she prioritizes environmental values when acting as a consumer.

Lifestyle and behavior factors explain more of the variation in the dependent variable than do the environmental beliefs. Here we find that those individuals who report more frequent lifestyle activities based on either efforts toward the greater good—i.e., volunteering, helping the environment, or spiritual practices—or personal advancement through professional or educational development, tend to be willing to pay more for sustainable beer. Similarly, those who already practice sustainable consumption behaviors such as composting, or buying organic, recycled, or local products are also willing to pay a higher price for sustainable beer. These findings suggest, more broadly, that those who already exhibit sustainable consumption behavior and pursue a lifestyle exhibiting higher civic and/or other social engagement are the individuals who will embrace sustainability offerings from brewers.

An individual’s beer preferences are especially important in predicting a WTP premium for sustainable beer. The beer preference category accounts for almost half of the R-squared decomposition. Results reveal that the frequency by which a respondent purchases or consumes a beer, or where that beer is consumed, are statistically insignificant. Also, counter to theoretical expectations, we find that respondents who consume exclusively American lagers are no different than individuals that consume other types of beer in their average reported WTP, although this variable is statistically significant in Model 4 at the ten percent significance threshold. A particularly important predictor of WTP for sustainable beer is the amount that one already pays for the non-sustainable version of that same beer. For each additional $1.00 that one pays per ounce of beer, an individual is willing to pay a premium of 7.4 cents per ounce for the sustainable version.

Personal demographics only account for about 9% of the R-squared decomposition and, as a variable grouping are statistically insignificant. Those who are younger and those with lower levels of education are willing to pay more for sustainable beer. Contrary to other studies’ findings about the importance of children or location of residence, we find no evidence that these conditions matter.

## Discussion and conclusion

In this analysis, we sought to address the question of whether consumers are willing to pay a premium for beer that is brewed with sustainable practices and, if so, which factors are associated with a consumers’ WTP. Results of a survey of beer consumers across the U.S. and a statistical analysis revealed that the majority of consumers are willing to pay more for sustainable beer. Consumers who are willing to pay a premium for sustainable beer tend to be more aware of their purchasing behavior, their responsibilities as both consumers and stewards of the Earth, and the product offerings that are available to them. They hold themselves and the producers responsible for sustainability decisions. These individuals also have routinized consumption behaviors and pursue lifestyles based on professional advancement, helping the environment, and helping other causes.

One of the most important predictors of WTP was the amount that consumers already pay for their ideal beer. This finding suggests that consumers who already highly value the quality or other attributes of the product, and pay more for these attributes, are also more inclined to pay an additional premium for sustainability. However, even if they are willing to pay a premium for sustainable attributes, these consumers still consider and balance other attributes of the commodity, such as price, quality, and brand image, among others [48]. It is important, therefore, that sustainability practices not compromise the quality or consistency of the product, or introduce functional risks, which previous studies have found to diminish interest in sustainable products [12, 13].

In addition, our results revealed that the structure of the WTP between the two tracks did not matter. While this does not altogether rule out the possibility of hypothetical bias in our data, in the absence of real consumer transactional data that may serve as “true” WTP values, methodologically, our finding suggests that at least in the context of similar open-ended WTP measures, non-ignorable variance is not always present. In other words, assuming high WTP variance suggests survey design problems that are at least in part rooted in hypothetical bias, the negative significance test between the two tracks in our study is salutary for the reliability of our estimates.

We note, however, that the bias may yet be present insofar as respondents take both WTP1 and WTP2 exercises to be equally hypothetical. For instance, the WTP1 measure was based on a current preferred beer, which may depend on respondents’ beliefs about how likely that particular beer is to be produced sustainably. However, an exercise that imagines a hypothetical product and then adds another counterfactual layer of sustainable practice on top of it (WTP2) would seem at least prima facie even less likely to turn into reality–and yet there was no difference between the two constructs. This also suggests that beer producers could either transform currently available products into sustainable counterparts or devise entirely new sustainable product lines. But while there were no statistically significant differences in reported WTP, of course there may be production cost implications between the two.

We also note again that our data are based on a convenience sample derived from online crowdsourcing. Our sample differs from the general population along several demographic variables; and the results presented herein are specific to our MTurk sample of beer consumers. In addition, certain estimates may not reflect true population values if there is revealed to be a strong relation between propensity to join MTurk and either purchasing alcohol or adopting sustainable lifestyle behaviors. Personal demographics were not predictive of reported WTP in our study, but further research in this area is needed, particularly as the market for sustainable products continues to expand.

In this analysis, we found that consumers on average are willing to pay more for sustainably brewed beer. We conclude with two challenges related to this finding for future research. First, an important next step is to determine how the WTP per unit of beer translates into specific brewery investments in more efficient and low-carbon technologies. Second, given that one likely cannot taste the difference between a sustainable beer and a non-sustainable beer, we should consider how information about sustainable offerings can be conveyed to consumers, perhaps through eco-labels [10] or marketing. Studies based on eye tracking [49, 48] suggest that such information provision to consumers may make a difference for the uptake of sustainable or eco-commodities.

## Supporting information

S1 File Willingness-to-pay for sustainable beer survey instrument. (PDF)

S2 File Application of survey weights. (PDF)

S3 File Survey data. (XLSX)
