# Supplementary material for: Willingness-to-pay for sustainable beer
Source: PLoS One. 2018 Oct 5;13(10):e0204917. doi: 10.1371/journal.pone.0204917 (PMC6173403; doi:10.1371/journal.pone.0204917)
Supplement: S1 File — (PDF) [file pone.0204917.s001.pdf]

S1. Willingness-to-pay for sustainable beer survey instrument

What is your age?
If What is your age? Is Less Than 21, Then Skip To End of Block

In which U.S. state do you currently live?

- I do not live in the United States (1)
- Alabama (2)
- Alaska (3)
- Arizona (4)
- Arkansas (5)
- California (6)
- Colorado (7)
- Connecticut (8)
- Delaware (9)
- Florida (10)
- Georgia (11)
- Hawaii (12)
- Idaho (13)
- Illinois (14)
- Indiana (15)
- Iowa (16)
- Kansas (17)
- Kentucky (18)
- Louisiana (19)
- Maine (20)
- Maryland (21)
- Massachusetts (22)
- Michigan (23)
- Minnesota (24)
- Mississippi (25)
- Missouri (26)
- Montana (27)
- Nebraska (28)
- Nevada (29)
- New Hampshire (30)
- New Jersey (31)
- New Mexico (32)
- New York (33)
- North Carolina (34)
- North Dakota (35)
- Ohio (36)
- Oklahoma (37)
- Oregon (38)
- Pennsylvania (39)
- Rhode Island (40)
- South Carolina (41)
- South Dakota (42)
- Tennessee (43)
- Texas (44)
- Utah (45)
- Vermont (46)
- Virginia (47)
- Washington (48)
- Washington, District of Columbia (49)
- West Virginia (50)
- Wisconsin (51)

- Wyoming (52)

If I do not live in the United... Is Selected, Then Skip To End of Block

On average, how frequently do you drink one or more beers in a day?

- 4 times a week or more (1)
- 1-3 times a week (2)
- 1-3 times a month (3)
- 1 time every other month or less (4)
- Never (5)

If 1 time every other month or... Is Selected, Then Skip To End of BlockIf Never Is Selected, Then Skip To End of Block

Considering the beer that you drink, about how often are you the one who actually buys the beer?

- Always (1)
- Usually (2)
- Sometimes (3)
- Rarely (4)
- Never (5)

If Rarely Is Selected, Then Skip To End of BlockIf Never Is Selected, Then Skip To End of Block

About how many beers have you consumed in the past week?

Where do you most often drink beer?

- Home (1)
- Restaurants (2)
- Bars (3)
- Brewpubs or breweries (4)
- Parties, gatherings, or other social events (5)
- Other (please specify) (6) ____________________

Which style(s) of beer do you buy most often? (Select up to three.)

- Light/pale American lager (1)
- Dark lager (2)
- Pilsner (3)
- Pale ale (4)
- India Pale Ale (IPA) (5)
- Dark ale (6)
- Stout (7)
- Bock (8)
- Wheat (9)
- Barleywine (10)
- Scottish ale (11)
- Porter (12)
- Belgian (13)
- Other (please specify) (14) ____________________

Out of the total amount of beer that you buy, please identify how frequently you purchase each of the following types.

	Always (1)	Usually (2)	Sometimes (3)	Rarely (4)	Never (5)
Domestic craft beer (1)	❍	❍	❍	❍	❍
Domestic mass-produced beer (2)	❍	❍	❍	❍	❍
Imported beer (including either craft or mass-produced) (3)	❍	❍	❍	❍	❍

How often do you brew your own beer (i.e. home brew)?
❍ Several times a year (1)
❍ A few times a year (2)
❍ About once a year (3)
❍ Just tried it once or twice (4)
❍ Never (5)

When you buy beer at the store, how much do you usually spend on a 6-pack?
❍ $3.00 - $5.99 (1)
❍ $6.00 - $7.99 (2)
❍ $8.00 - $9.99 (3)
❍ $10.00 - $12.00 (4)
❍ I do not buy beer in a 6-pack (5)

Imagine yourself standing at the store in front of a full shelf of different beer options, and you are looking to buy one of them. Besides cost, what factor do you think would most influence the decision that you make about which to buy?
❍ Something new that I have never had before (1)
❍ Graphics and visuals on the packaging (2)
❍ Reputation of the brewery (3)
❍ If the beer is a favorite (4)
❍ If a beer is local (5)
❍ Word of mouth from others about what is good (6)
❍ Other (please specify) (7) ____________________

Randomly assign respondents into WTP1 or WTP2

WTP1
Please think about a beer that you really like to drink. In the next few questions, we will ask you to describe a few things about this particular beer.

What is the brand and name of this beer?

SizePackage What size package does this preferred beer come in?
- ❍ single beer at a time (1)
- ❍ 4-pack (2)
- ❍ 6-pack (3)
- ❍ 12-pack (4)
- ❍ 24-pack (5)
- ❍ other (6) ____________________

Approximately how much is a ${q://QID76/ChoiceGroup/SelectedChoicesTextEntry} of this beer? Please enter the price in dollars and cents.

Many breweries across the U.S. are investing in equipment that helps them conserve energy or water, or use an electricity source that produces limited greenhouse gas emissions, such as solar panels. For consumers, these practices could make these beers more expensive, but with the benefit of saving energy and reducing greenhouse gas emissions.

If ${q://QID48/ChoiceTextEntryValue}${q://QID57/ChoiceTextEntryValue} were brewed using such practices, would you be willing to pay more than $${q://QID55/ChoiceTextEntryValue} for a ${q://QID76/ChoiceGroup/SelectedChoicesTextEntry} of it?
- ❍ No (1)
- ❍ Yes (2)

Display This Question:
If If ${q://QID48/ChoiceTextEntryValue}${q://QID57/ChoiceTextEntryValue} were brewed using such prac... Yes Is Selected

WTPamt1 How much more would you be willing to pay for a ${q://QID76/ChoiceGroup/SelectedChoicesTextEntry} of this beer in addition to $${q://QID55/ChoiceTextEntryValue}? Please enter the maximum additional amount in dollars and cents.

If a beer were brewed sustainably, would you be more or less likely to buy it over other similar beers, assuming no change in price?
- ❍ Much more likely (1)
- ❍ Somewhat more likely (2)
- ❍ Neither more nor less likely (3)
- ❍ Somewhat less likely (4)
- ❍ Much less likely (5)

WTP2
Please imagine a beer you would most enjoy drinking - think of it as your "ideal" beer. In the next few questions, we will ask you to describe a few things about this beer.

For your ideal beer, rank the color from 1 to 10, where 1 is very light or pale and 10 is very dark. Please answer to the best of your ability, even if you think you don't know much about beer in general.

- Enter a number between 1 and 10. (1) ____________________
- Don't care about the color (2)

For your ideal beer, rank how hoppy the beer is from 1 to 10, where 1 is no flavor of hops at all and 10 is extremely hoppy. Please answer to the best of your ability, even if you think you don't know much about beer in general.

- Enter a number between 1 and 10. (1) ____________________
- Don't care about the hoppiness (2)

For your ideal beer, rank how malty the beer is from 1 to 10, where 1 is no flavor of malt at all and 10 is extremely malty. Please answer to the best of your ability, even if you think you don't know much about beer in general.

- Enter a number between 1 and 10. (1) ____________________
- Don't care about the maltiness (2)

What size package would this ideal beer come in at the store?

- single beer at a time (1)
- 4-pack (2)
- 6-pack (3)
- 12-pack (4)
- 24-pack (5)
- other (please specify) (6) ____________________

Please tell us any other details about this beer that you would like to share.

If you found this exact beer on the shelf at a store, what is the most you would pay for a ${q://QID77/ChoiceGroup/SelectedChoicesTextEntry} of it? Please enter this price in dollars and cents.

Many breweries across the U.S. are investing in equipment that helps them conserve energy or water, or use an electricity source that produces limited greenhouse gas emissions, such as solar panels. For consumers, these practices could make these beers more expensive, but with the benefit of saving energy and reducing greenhouse gas emissions.

If your ideal beer were brewed using such practices, would you be willing to pay more than $${q://QID43/ChoiceTextEntryValue} for a ${q://QID77/ChoiceGroup/SelectedChoicesTextEntry} at a store?

- No (1)
- Yes (2)

Display This Question:

If If your ideal beer were brewed using such practices, would you be willing to pay more than $... Yes Is Selected

WTPamt2 How much more would you be willing to pay for a ${q://QID77/ChoiceGroup/SelectedChoicesTextEntry} of this beer at the store in addition to $${q://QID43/ChoiceTextEntryValue}? Please enter the maximum additional amount.

If a beer were brewed sustainably, would you be more likely to buy it over other similar beers, assuming no change in price?

- ❍ Much more likely (1)
- ❍ Somewhat more likely (2)
- ❍ Neither more nor less likely (3)
- ❍ Somewhat less likely (4)
- ❍ Much less likely (5)

Please tell us which aspects you value most in a brewery. (Select all that apply.)

- ❑ Whether it’s local (1)
- ❑ Sustainability practices (2)
- ❑ Community involvement (3)
- ❑ Independently owned (4)
- ❑ Long history or tradition (5)
- ❑ Other (please specify) (7) ____________________
- ❑ I don’t go to breweries (8)
- ❑ Value-conscious (6)

How knowledgeable would you say you are about types of beer, as well as beer in general?

- ❍ Very knowledgeable (1)
- ❍ Somewhat knowledgeable (2)
- ❍ Not too knowledgeable (3)
- ❍ A little knowledgeable (4)
- ❍ I haven't really thought much about this topic (5)

We will now ask you about a series of activities. For each one, please identify how frequently you engage in that activity.

	Very frequently (1)	Frequently (2)	Occasionally (3)	Rarely (4)	Never (5)
Developing your career (1)	❍	❍	❍	❍	❍
Playing sports, exercise, or recreation (2)	❍	❍	❍	❍	❍
Helping the environment (3)	❍	❍	❍	❍	❍
Enjoying nature and the outdoors (4)	❍	❍	❍	❍	❍
Religious or spiritual practices (5)	❍	❍	❍	❍	❍
Researching or trying new technology (6)	❍	❍	❍	❍	❍
School, lectures, or other education (7)	❍	❍	❍	❍	❍
Shopping (8)	❍	❍	❍	❍	❍
Socializing with others (9)	❍	❍	❍	❍	❍
Taking care of/spending time with family (10)	❍	❍	❍	❍	❍
Using the Internet for fun or leisure (11)	❍	❍	❍	❍	❍
Watching TV or movies (12)	❍	❍	❍	❍	❍
Volunteering or donating to charity (13)	❍	❍	❍	❍	❍

Generally speaking, how concerned are you about environmental issues? Please tell me what you think, where 1 means you are not at all concerned and 5 means you are very concerned.

- ❍ 1 Not at all concerned (1)
- ❍ 2 (2)
- ❍ 3 (3)
- ❍ 4 (4)
- ❍ 5 Very concerned (5)

In general, I purchase environmentally-friendly or “green” products when the option is available to do so.

- ❍ Always (1)
- ❍ Usually (2)
- ❍ Sometimes (3)
- ❍ Rarely (4)
- ❍ Never (5)

We will now ask you some questions about different types of activities. For each item, mark how frequently you engage in that behavior.

	Always (1)	Usually (2)	Sometimes (3)	Rarely (4)	Never (5)
Compost waste? (1)	❍	❍	❍	❍	❍
Use your own bag instead of a plastic or paper one provided by a store? (2)	❍	❍	❍	❍	❍
Purchase organically grown food? (3)	❍	❍	❍	❍	❍
Purchase recycled paper products (such as toilet paper and writing paper)? (4)	❍	❍	❍	❍	❍
Turn off the water faucet while brushing your teeth? (5)	❍	❍	❍	❍	❍
Keep heating low to save energy? (6)	❍	❍	❍	❍	❍
Recycle? (7)	❍	❍	❍	❍	❍
Buy from local stores? (8)	❍	❍	❍	❍	❍

Please rank the degree to which you agree with the following statements.

My choices as a consumer will have a direct impact on the environment.
- Strongly agree (1)
- Agree (2)
- Somewhat agree (3)
- Neither agree nor disagree (4)
- Somewhat disagree (5)
- Disagree (6)
- Strongly disagree (7)

Companies have the responsibility to make all of their products more environmentally-friendly.
- Strongly agree (1)
- Agree (2)
- Somewhat agree (3)
- Neither agree nor disagree (4)
- Somewhat disagree (5)
- Disagree (6)
- Strongly disagree (7)

For the following statements, indicate the degree to which you agree or disagree.

	Strongly agree (1)	Agree (2)	Somewhat agree (3)	Neither agree nor disagree (4)	Somewhat disagree (5)	Disagree (6)	Strongly disagree (7)
There are no limits to growth for nations like the United States. (1)	❍	❍	❍	❍	❍	❍	❍
Modifying the environment seldom causes serious problems. (2)	❍	❍	❍	❍	❍	❍	❍
Science will help us to live without conservation. (3)	❍	❍	❍	❍	❍	❍	❍
Humans were created to rule over nature. (4)	❍	❍	❍	❍	❍	❍	❍
The balance of nature is delicate and easily upset. (5)	❍	❍	❍	❍	❍	❍	❍
The Earth is like a space ship, with limited room and resources. (6)	❍	❍	❍	❍	❍	❍	❍
Plants and animals do not exist primarily for human use. (7)	❍	❍	❍	❍	❍	❍	❍
One of the most important reasons for conservation is to preserve	❍	❍	❍	❍	❍	❍	❍

wild areas. (8)							
Technology will solve many environmental problems. (9)	❍	❍	❍	❍	❍	❍	❍
Exploitation of resources should be stopped. (10)	❍	❍	❍	❍	❍	❍	❍

We will now ask you a series of questions about demographics and technology use.

What is your gender?
❑ Male (1)
❑ Female (2)

What is your race? (Select all that apply.)
❑ White (1)
❑ Black or African American (2)
❑ American Indian or Alaska Native (3)
❑ Asian (4)
❑ Native Hawaiian or Pacific Islander (5)
❑ Other (please specify) (6) ____________________

What is your marital status?
❍ Married (1)
❍ Living as married (2)
❍ Widowed (3)
❍ Divorced (4)
❍ Separated (5)
❍ Single, never been married (6)

How many people, including any children, live in your household?

If you have ever owned or currently own a tablet computer (for example, iPad, Samsung Galaxy Tab, Google Nexus, Kindle Fire, etc.), approximately when did you acquire your first tablet?
❍ This year (in 2016) (1)
❍ Last year (in 2015) (2)
❍ About 2 years ago (in 2014) (3)
❍ About 3 or 4 years ago (in 2013 or 2012) (4)
❍ About 5 or 6 years ago (in 2011 or 2010) (5)
❍ Longer than 6 years ago (in 2009 or earlier) (6)
❍ I have never owned a tablet computer (7)

When it comes to politics do you usually think of yourself as extremely liberal, liberal, slightly liberal, moderate or middle of the road, slightly conservative, conservative, extremely conservative, or haven't you thought much about this?

- Extremely liberal (1)
- Liberal (2)
- Slightly liberal (3)
- Moderate or middle of the road (4)
- Slightly conservative (5)
- Conservative (6)
- Extremely conservative (7)
- I haven't thought much about this (8)

What is the highest level of education you have completed?

- Less than high school (1)
- High school / GED (2)
- Some college (3)
- 2-year college degree (4)
- 4-year college degree (5)
- Master's degree (6)
- Professional degree (JD, MD) (7)
- Doctoral degree (8)

Considering all sources, which of the following best describes your total household income before taxes in 2015?

- Under $15,000 (1)
- $15,000 to $24,999 (2)
- $25,000 to $34,999 (3)
- $35,000 to $49,999 (4)
- $50,000 to $74,999 (5)
- $75,000 to $99,999 (6)
- $100,000 to $149,999 (7)
- $150,000 to $199,999 (8)
- $200,000 to $249,000 (9)
- Above $250,000 (10)

How would you describe your area of residence?

- Rural (1)
- Urban (2)
- Suburban (3)
- Other (please specify) (4) ____________________
