# Supplementary material for: Willingness-to-pay for sustainable beer
Source: PLoS One. 2018 Oct 5;13(10):e0204917. doi: 10.1371/journal.pone.0204917 (PMC6173403; doi:10.1371/journal.pone.0204917)
Supplement: S2 File — (PDF) [file pone.0204917.s002.pdf]

S2. Application of survey weights

While our primary focus in this analysis is on a beer consumer population, which we believe we were effectively able to recruit through an MTurk sample, it is additionally insightful to consider how results may change if our sample more closely aligned with the demographics—age, education, and gender—of the American public. Thus, in this Supporting Information section, we apply a survey weight accordingly and re-estimate the regression model. S2 Table 1 presents these results.

The weighted results are mostly consistent with the main results, with only a few exceptions. Gender is no longer statistically significant in Model 5 and age is no longer significant in the combined model, Model 6. Those who believe that companies should make their products more eco-friendly are no less likely to pay more for sustainable beer in the combined Model 6. The only variable that suggests enhanced importance in these models, relative to the main results, is the growth limits and human science factor. Those that hold beliefs that there are few if any limits to growth, or that science and technology can solve environmental problems, are willing to pay less for sustainable beer than those that do not hold such beliefs.

Table A. Regression results with survey weights and dependent variable WTP for sustainable beer (in $/oz)

	Model 1	Model 2	Model 3	Model 4	Model 5	Model 6
WTP Track						
WTP1	0.00153					0.00000965
	(0.00264)					(0.00249)
**Environmental Beliefs**						
Delicate environment and resource constraints		-0.00239				-0.000796
		(0.00178)				(0.00189)
Growth limits and human science		-0.00159				-0.00192**
		(0.00997)				(0.000767)
Consumption affects the environment		0.00286***				0.00141*
		(0.00101)				(0.000967)
Companies are responsible		0.00296***				0.000747
		(0.000976)				(0.000967)
**Lifestyle and Behavior**						
Social and leisure			0.00180			0.00201
			(0.00198)			(0.00164)
Greater good			0.00145			0.00245*
			(0.00130)			(0.00140)
Advancement			0.00129			0.00249**
			(0.00108)			(0.00104)
Sports and nature			0.000440			0.000491
			(0.00102)			(0.000998)
Green consumption			0.00600***			0.00468***
			(0.00104)			(0.00121)
Conservation and recycling			0.00228**			0.00127
			(0.00123)			(0.000997)
**Beer Preferences**						
Purchase frequency				0.00229		0.00233
				(0.00182)		(0.00213)
Number of beers				-0.0000782		-0.0000759
				(0.0000880)		(0.0000846)
House				-0.00373		-0.00436*
				(0.00264)		(0.00259
American lager consumer				-0.00233		-0.00253
				(0.00227)		(0.00269)
Price per ounce				0.0729***		0.0718***
				(0.0225)		(0.0215)
**Personal Demographics**						
Age					-0.000177*	-0.000118
					(0.000107)	(0.0000965)
Male					-0.00338	-0.000189
					(0.00259)	(0.00278)
Married or equivalent					0.00298	0.00276
					(0.00284)	(0.00263)
Number in household					0.000717	0.00149
					(0.00110)	(0.00121)
Political leaning					-0.00159**	0.000264
					(0.000694)	(0.000767)
Education					-0.00124	-0.00227**
					(0.00116)	(0.000937)
Income					-0.000494	-0.00151
					(0.00103)	(0.00126)
Rural					-0.000895	0.00128
					(0.00438)	(0.00384)
Urban					0.000172	0.00197
					(0.00924)	(0.00277)
Constant	0.0160***	-0.0115***	0.0168***	0.00579	0.0346***	0.00693
	(0.00139)	(0.00655)	(0.00123)	(0.00431)	(0.00924)	(0.0101)
	1094	1061	1056	1094	1066	1000

Standard errors in parentheses; * p<0.10, * p<0.05, *** p<0.01
